# Supplementary material for: Metaplastic and energy-efficient biocompatible graphene artificial synaptic transistors for enhanced accuracy neuromorphic computing
Source: Nat Commun. 2022 Jul 28;13:4386. doi: 10.1038/s41467-022-32078-6 (PMC9334620; doi:10.1038/s41467-022-32078-6)
Supplement: Supplementary file 1 — Supplementary Information [file 41467_2022_32078_MOESM1_ESM.pdf]

SUPPLEMENTARY INFORMATION

FOR

METAPLASTIC AND ENERGY-EFFICIENT BIOCOMPATIBLE GRAPHENE ARTIFICIAL SYNAPTIC TRANSISTORS FOR ENHANCED ACCURACY NEUROMORPHIC COMPUTING

Dmitry Kireev[1,2]*, Samuel Liu[1]*, Harrison Jin[1], T. Patrick Xiao[3], Christopher H. Bennett[3], Deji Akinwande[1,2], and Jean Anne C. Incorvia[1,2]

[1] Department of Electrical and Computer Engineering, The University of Texas at Austin, Austin, TX, USA

[2] Microelectronics Research Center, The University of Texas, Austin, Texas, 78758 USA

[3] Sandia National Laboratories, Albuquerque, New Mexico 87123, USA

* these authors contributed equally

Supplementary Table 1. Comparing performance of our BLAST devices to other organic and 2D-material based neuromorphic systems. EC – electrochemical; ++ means high; + moderate; - low; * - means material is potentially toxic; N/R – not reported.

Device: Material	Device: Secondary	Type	Speed, sec	Energy, J/μm2	Endurance	Write noise	Linearity	On/Off Ratio
BLAST (this work) Graphene	Nafion (13 mm^2)	EC	10^{-4}	$3.3x10^{-17}$	$>10^7$	1.92%	1.03/0.74 ++	3.51
	Nafion (42 mm^2)	EC	10^{-4}	$4.7x10^{-18}$	$>10^7$	2.21%	0.66/0.56 ++	N/R
	Nafion (85 mm^2)	EC	10^{-4}	$4.8x10^{-17}$	$>10^7$	1.03%	0.33/0.24 ++	N/R
	Nafion (400 um^2)	EC	10^{-4}	$1.4x10^{-15}$	N/R	2.01%	1.79/0.29 +	12.5
PEDOT:PSS[1]	Nafion	EC	10^{-2}	$4x10^{-16}$	N/R	<1% –	++	3.7
PEDOT:PSS[2]	TFSI(PVDF-HFP)	EC	$2.5x10^{-4}$	$4x10^{-15}$	N/R	0.023% –	0.70/-0.12 ++	1.2
$RbAg_4I_5$[3]	MEH-PPV	EC	$2x10^{-3}$	$3.3x10^{-13}$	N/R	N/R	N/R	N/R
MoS_2[4]	hBN	Charge trap	10^{-3}	$2.7x10^{-16}$	N/R	Moderate	Moderate	$\sim10^5$
BP[5]	POx *	Charge trap	10^{-2}	$8x10^{-10}$	N/R	High	Low	1.2
Graphene[6]	MoS_2+light	Charge trap	$5x10^{-2}$	$1.7x10^{-12}$	N/R	Low	Low	500
MoS_2[7]	PTCDA	Charge trap	$2.5x10^{-2}$	$9.4x10^{-13}$	N/R	High	Low	167
MoS_2[8]	hBN	Charge trap	10^{-1}	$6x10^{-15}$	$>10^5$	High	Low	8.1
Graphene[9]	$LiClO_4$-PEO*	EC	10^{-2}	$8.3x10^{-15}$	>500	Low	Moderate	11
WSe_2[10]	$LiClO_4$-PEO*	EC	10^{-1}	10^{-14}	N/R	Moderate	High	2.2
MoO_3[11]	$LiClO_4$-PEO*	EC	10^{-2}	$1.8x10^{-13}$	$>4x10^4$	7.9% Moderate	Moderate	1.7
MoS_2[12]	---	EC	10^{-3}	$1.1x10^{-9}$	$>4x10^4$	High	Moderate	5
WO_3[13]	DEME-TFSI*	EC	$2x10^{-1}$	$1.4x10^{-15}$	N/R	Low	Low	7.2
MoS_2[14]	chitosan	EC	10^{-2}	$1.4x10^{-13}$	N/R	High	Low	3.5
Graphene[15]	Silver gel	EC	1	$3.1x10^{-10}$	N/R	Low	Low	5.6
ITO[16]	Maltose-ascorbic acid	EC	10^{-2}	N/R	420	Low	Low	3
NaAc doped PVA[17]	---	EC	10^{-1}	$1.32x10^{-16}$	>240	Low	Low	5
Graphdiyne[18]	Li-GAS/Na-GAS	EC	$4.4x10^{-1}$	$1.8x10^{-8}$	N/R	Low	Moderate	4
Cellulose nanocrystal[19]	Ag nanoparticles	EC	10^{-3}	$3x10^{-12}$	$>10^4$	High	Moderate	10^4
SnSe*[20]	NSTO	RRAM	$4x10^{-7}$	$8.2x10^{-15}$	N/R	High	Low	10^4
hBN[21]	---	RRAM	$2x10^{-5}$	$1.7x10^{-12}$	$>10^7$	N/A	N/A	16
WS_2[22]	ZrO_2	RRAM	$5x10^{-8}$	$1.9x10^{-14}$	$>10^9$	High	Low	100
MoS_2[23]	---	RRAM	10^{-3}	$5x10^{-18}$	N/R	High	Low	5
Zein[24]	---	RRAM	10^{-6}	$1.9x10^{-12}$	50	High	Low	100
MoS_2[25]	P(VDF-TrFE)	FET	$5x10^{-3}$	$4x10^{-17}$	$>10^7$	High	Low	100
Graphene[26]	P(VDF-TrFE)	FET	10^{-1}	10^{-9}	-	Low	Low	2.2
α-In_2Se_3 *[27]	Al_2O_3	FET	$2x10^{-2}$	$2x10^{-13}$	-	High	Low	100
Graphene[28]	Al_2O_3	FET	1	$1.3x10^{-2}$	>200	Moderate	High	1.5

Supplementary Note 1. Nonlinearity, read noise, and write noise calculation

To calculate nonlinearity, following the methodology outlined in Reference [29], the averaged ramp is normalized and a fitting is calculated for the following equations:

$$G_P = B\left(1 - e^{-P/A}\right) + G_{min} \tag{1a}$$

$$G_D = -B\left(1 - e^{\frac{P-P_{max}}{A}}\right) + G_{max} \tag{1b}$$

$$B = (G_{max} - G_{min})/\left(1 - e^{-P_{max}/A}\right) \tag{1c}$$

where G_P and G_D are the ramp values for potentiation and depression, G_{max} and G_{min} are the maximum and minimum conductance, and P_{max} is the maximum pulse number for the ramp. The fitting parameter A describes the nonlinearity of the ramp, where $A = 0$ is perfect linearity.

Write noise is calculated using the standard deviation of the averaged ramp, which is in turn averaged across all pulses:

$$\sigma_{pulse} = \sqrt{\left(\frac{\sum_{n=1}^{N}(|x-\bar{x}|)^2}{N}\right)} \tag{2a}$$

$$\sigma_{write} = \frac{mean(\sigma_{pulse})}{G_{max,avg} - G_{min,avg}} \tag{2b}$$

where x is a conductance sample, $\bar{x}$ is the mean of the samples, and N is the number of samples. $G_{max,avg}$ and $G_{min,avg}$ are the maximum and minimum conductance of the averaged ramp.

Read noise is calculated by first grouping all sampled data by conductance level and calculating the standard deviation as follows:

$$\sigma_{level} = \sqrt{\left(\frac{\sum_{n=1}^{N}(|x-\bar{x}|)^2}{N}\right)} \tag{3a}$$

where x is a conductance sample, $\bar{x}$ is the mean of the samples, and N is the number of samples. The average read noise across the dynamic range of the device is then calculated:

$$\sigma_{read} = \frac{mean(\sigma_{level})}{G_{max} - G_{min}} \tag{3b}$$

Supplementary Note 2. Energy dissipation per update calculation

Gate current and voltage are monitored for a current spike and the channel conductance before, during, and after the update are recorded. The raw energy dissipation can be obtained using:

$$E_{raw} = \int_{t_1}^{t_2} I_G \, V_G dt \tag{4}$$

where I_G and V_G are gate current and voltage respectively, and t_1 and t_2 denote the time window of the measurement.

To make the calculation generalizable across devices with different channel area, normalization is done according to device area:

$$E_{norm} = E_{raw}/A_{channel} \tag{5}$$

where $A_{channel}$ is the device channel area. In order to make sure that the value represents a similar change in conductance across all devices, a linear scaling law is assumed to scale all energy values to the energy required to change the conductance of the device by 1%, chosen since it is close to the write noise of the devices:

$$E_{norm}^{1p} = 0.01 \frac{G_{min}}{G_{max} - G_{min}} \mathrm{E_{norm}} \tag{6}$$

where G_{max} and G_{min} are the maximum and minimum channel conductances.

Supplementary Note 3. Read power dissipation

The read operation of the device depends on the channel conductance. As a result, the energy required for a read operation can be described as follows:

$$P_{read} = V_{read}^2 G \tag{7a}$$

$$E_{read} = P_{read} t_{read} \tag{7b}$$

where P_{read} is the read power, V_{read} is the applied read voltage, G is the channel conductance, and t_{read} is the read pulse length.

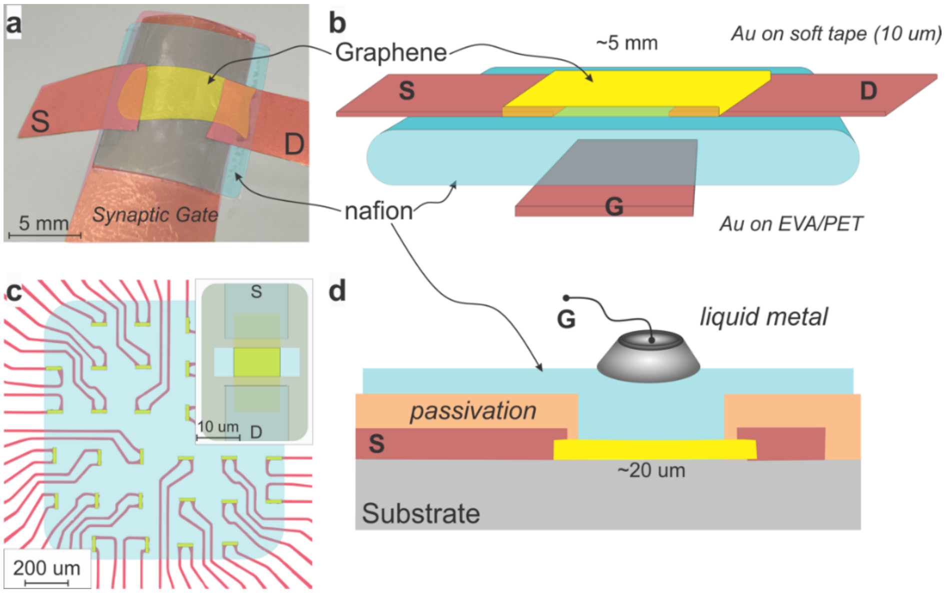

Supplementary Figure 1. Comprehensive schematic of mBLAST (a-b) and μBLAST (c-d) structures. **a**, artificially colored photograph of a mBLAST device. **b**, cross-section schematic of a MBLAST device. Nafion shown in cyan, graphene channel in yellow, and gold contacts in red. **c-d**, Schematic and cross-section of an array of 32 μBLASTs on a single chip. Color code is the same; passivation is shown in brown orange.

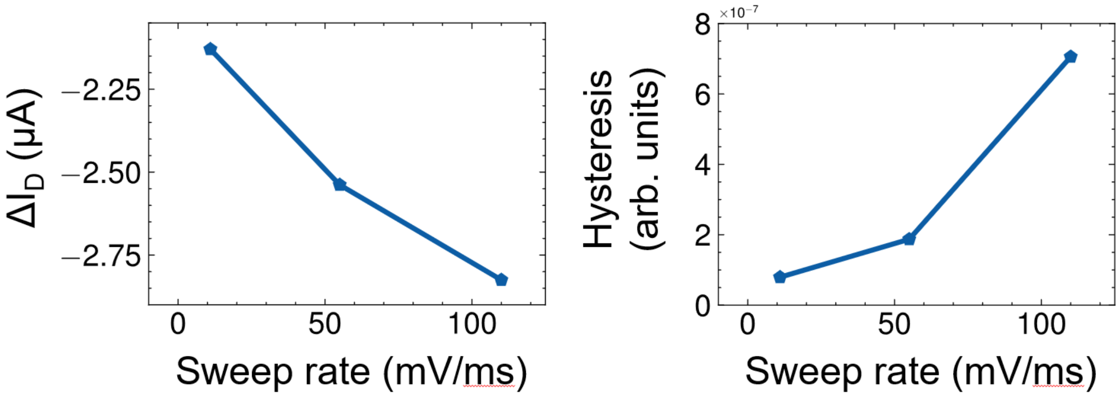

Supplementary Figure 2. a, Analysis of IV characteristics and **b**, its hysteresis. The hysteresis is defined as the overall area within the curve. Clearly faster sweep rate results in considerably larger hysteresis.

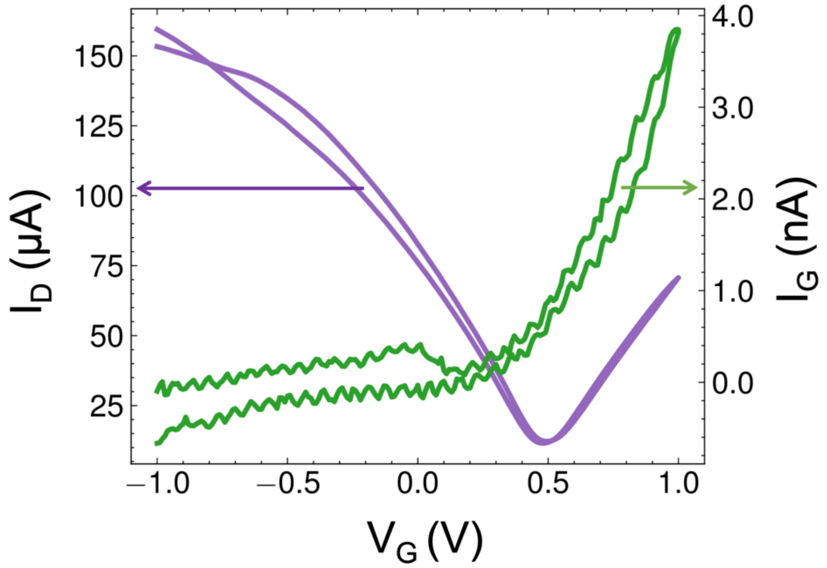

Supplementary Figure 3. Microscale BLAST device transfer characteristics. Gate current is shown in green and drain current is shown in purple.

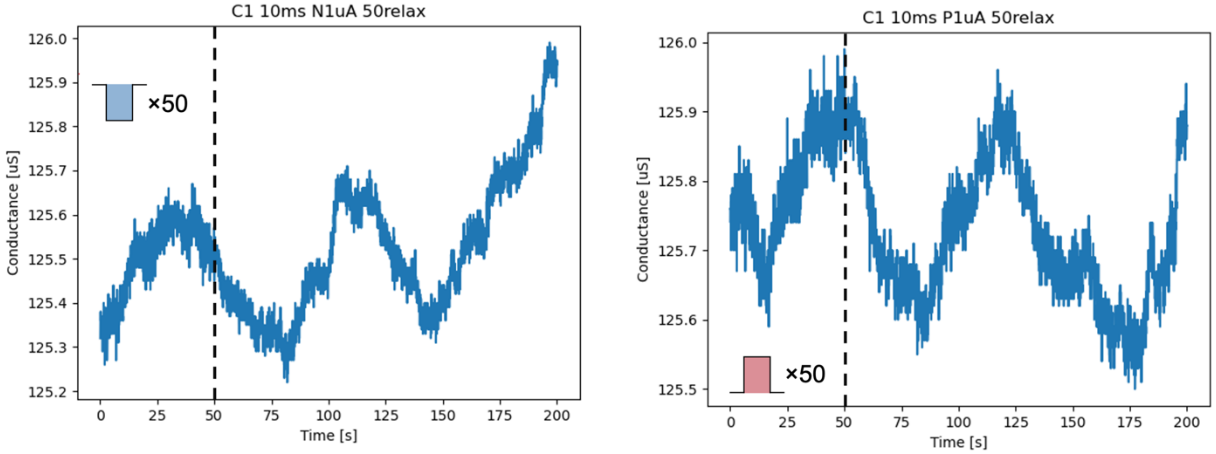

Supplementary Figure 4. Control experiments with semiconducting PEDOT:PSS instead of graphene. 50 current pulses (1 μA, 10 ms) are delivered to the device at a period of 1 s for **a**, negative polarity and **b**, positive polarity. Delivered current pulses do not cause appreciable conductance modulation.

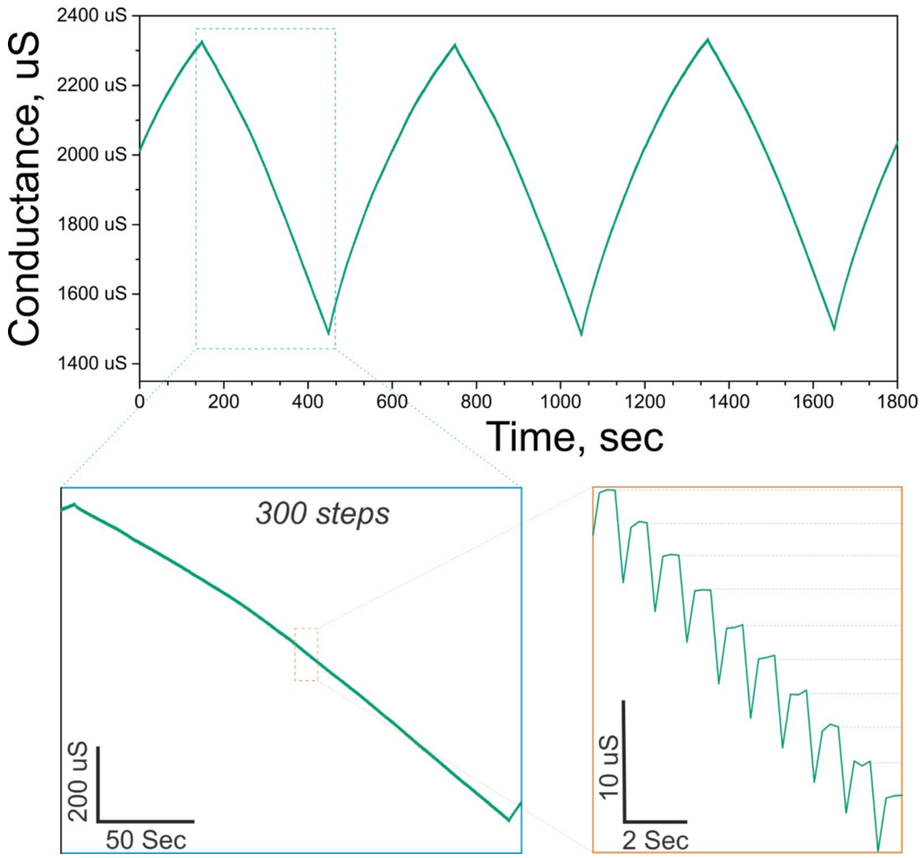

Supplementary Figure 5. 300 definitive steps ramp.

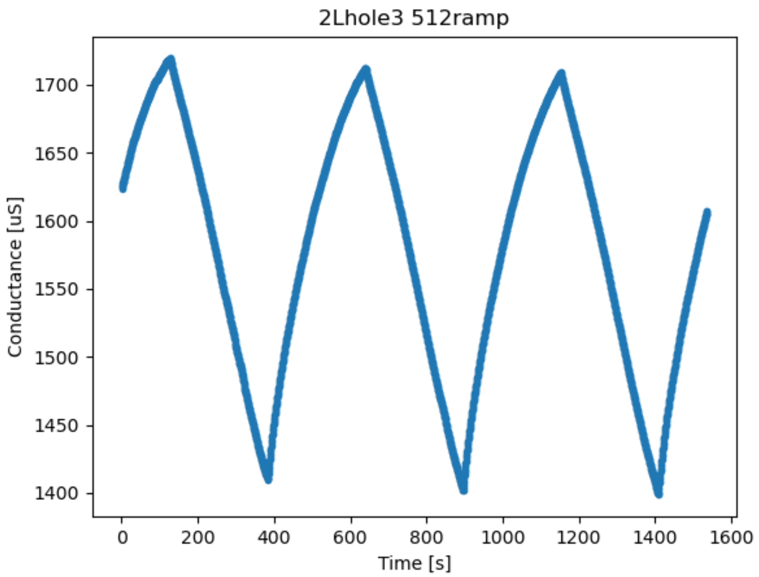

Supplementary Figure 6. 512 Steps per ramp.

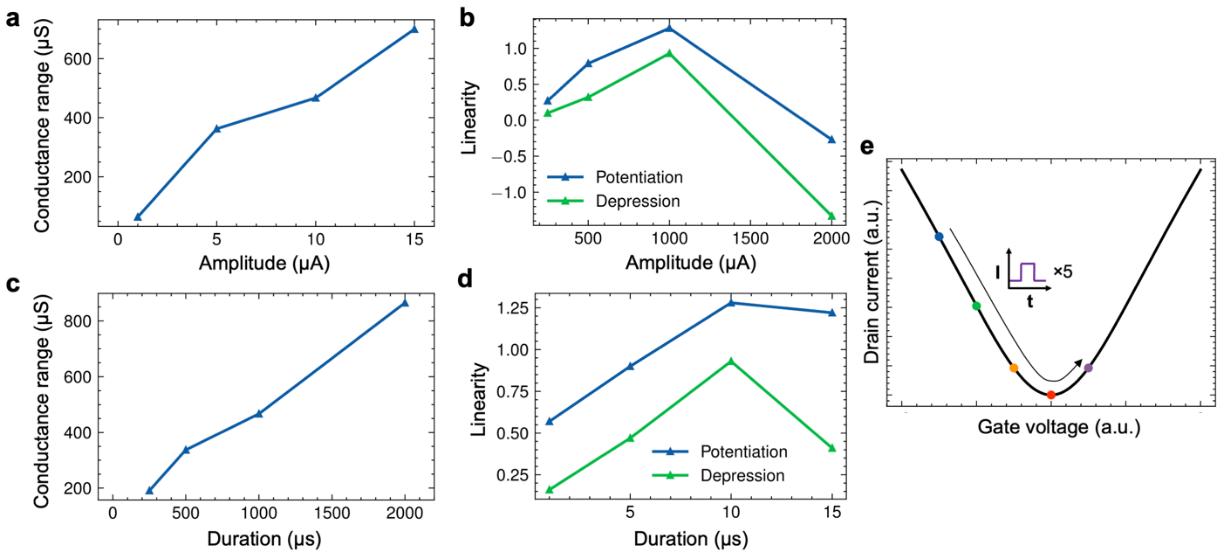

Supplementary Figure 7. Change in conductance range as a function of gate pulse amplitude, **a**, and duration, **c**, and linearity as a function of gate pulse amplitude, **b**, and duration, **d**. The dip in linearity at high duration and amplitude is due to the charge neutrality point being reached at high dynamic range. This causes a positive conductance change when additional pulses are applied, shown in **e**.

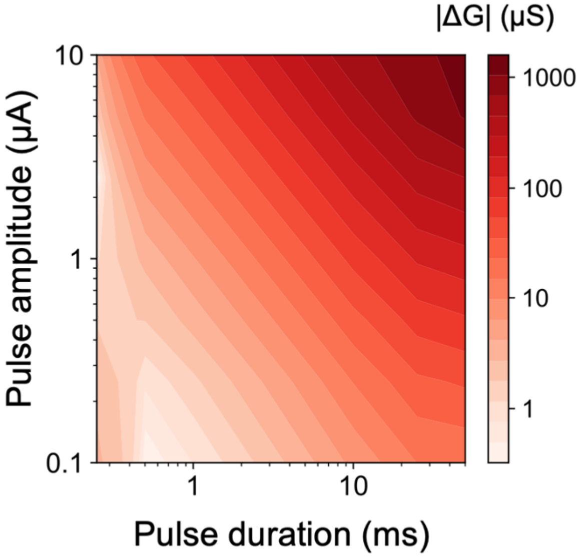

Supplementary Figure 8. Log-log plot of conductance change as a function both current pulse amplitude and duration. It is evident that ΔG is linearly dependent on both variables.

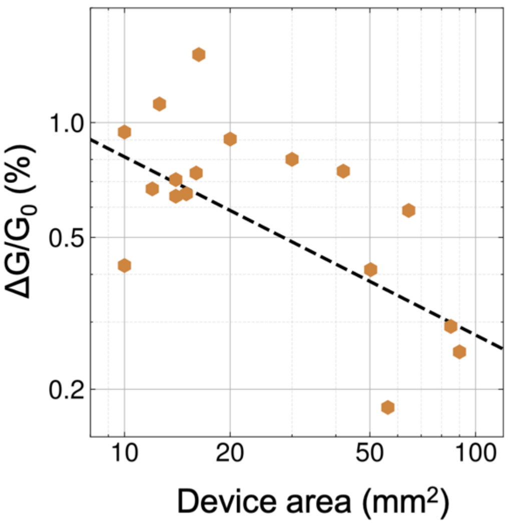

Supplementary Figure 9. Normalized conductance change as a function of device area for all mBLAST devices. There is a weakly decreasing trend ($R = -0.7$) as device area increases.

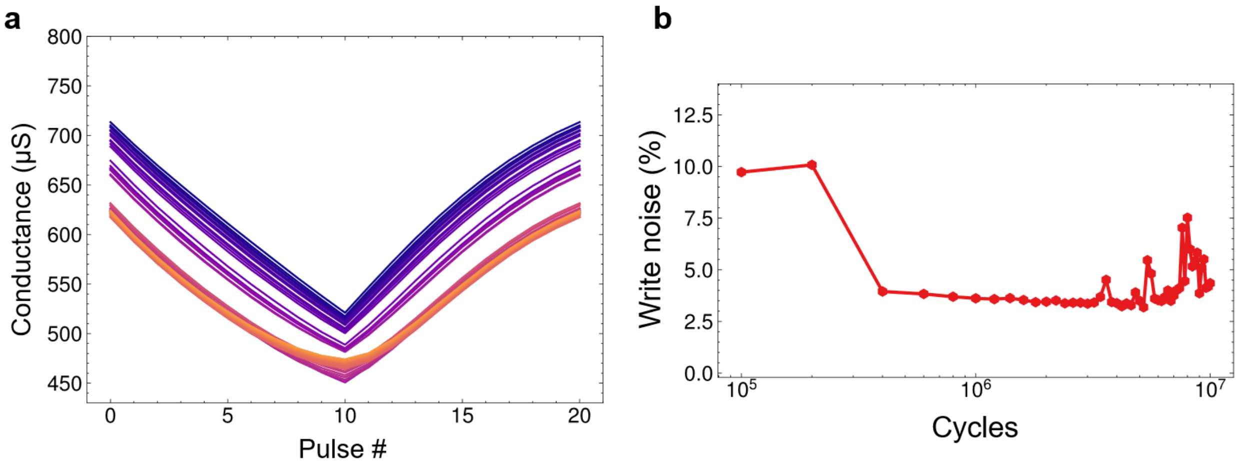

Supplementary Figure 10. a, 45 averaged ramps between 10^6 and 10^7 pulses at intervals of 2×10^5 pulses. Averaged ramp after 10^6 pulses is shown in dark blue and averaged ramp after 10^7 pulses is shown in orange, where ramps with intermediate colors represent intermediate values. **b,** Write noise as a function of pulse number.

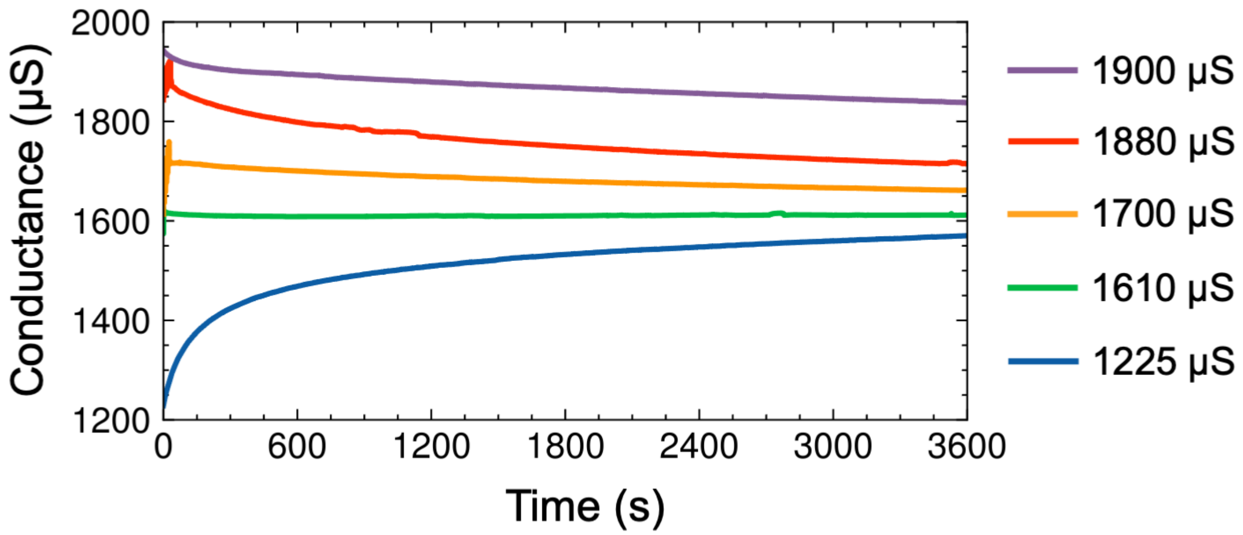

Supplementary Figure 11. Retention measurement of 5 states that start from different initial conductances in an mBLAST device for one hour. The initial conductance is indicated in the legend to the right. The green state is stable because it is the equilibrium point of the device.

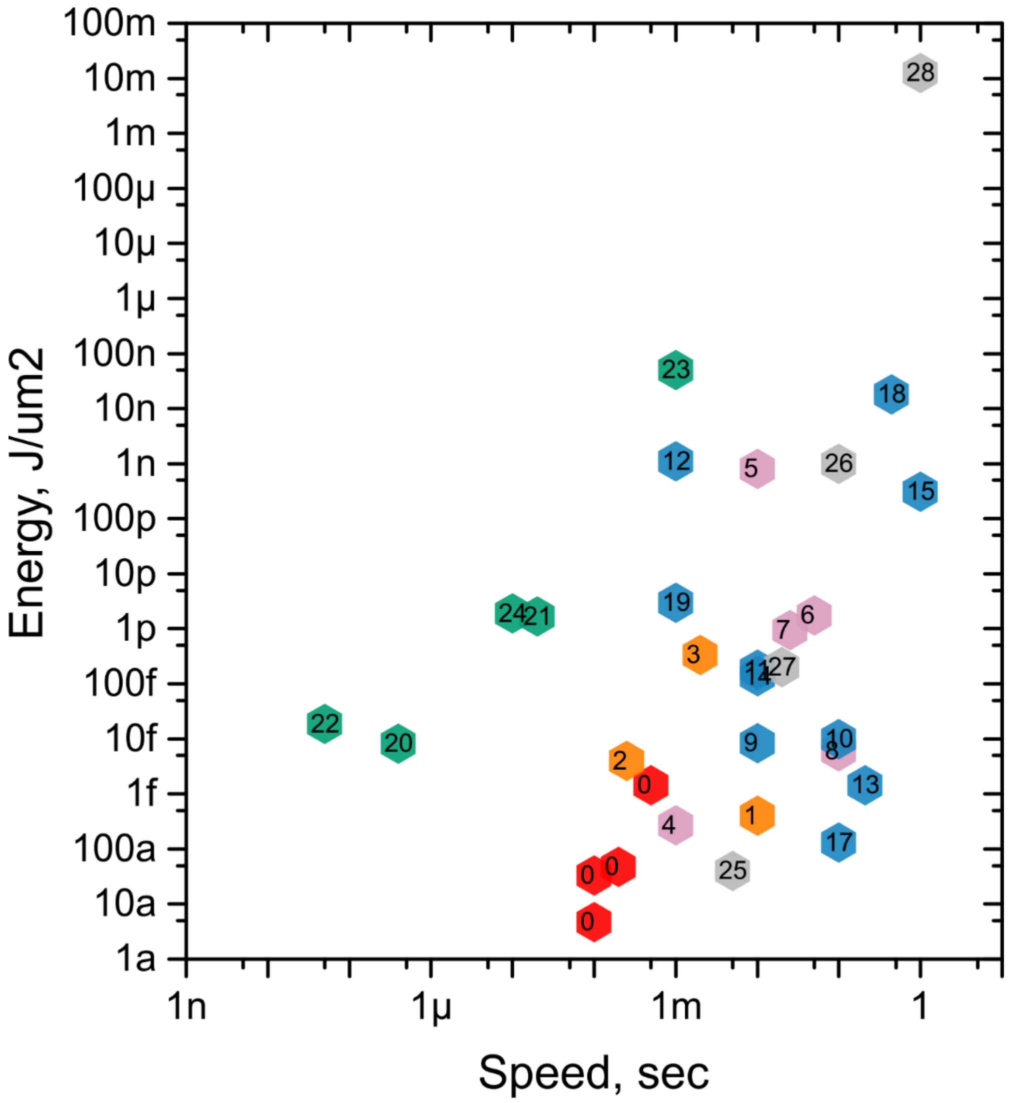

Supplementary Figure 12. Reference numbers corresponding to each datapoint presented in Fig. 3h in the main text and directly correlates to the supplementary Table S1. [0] corresponds to this work.

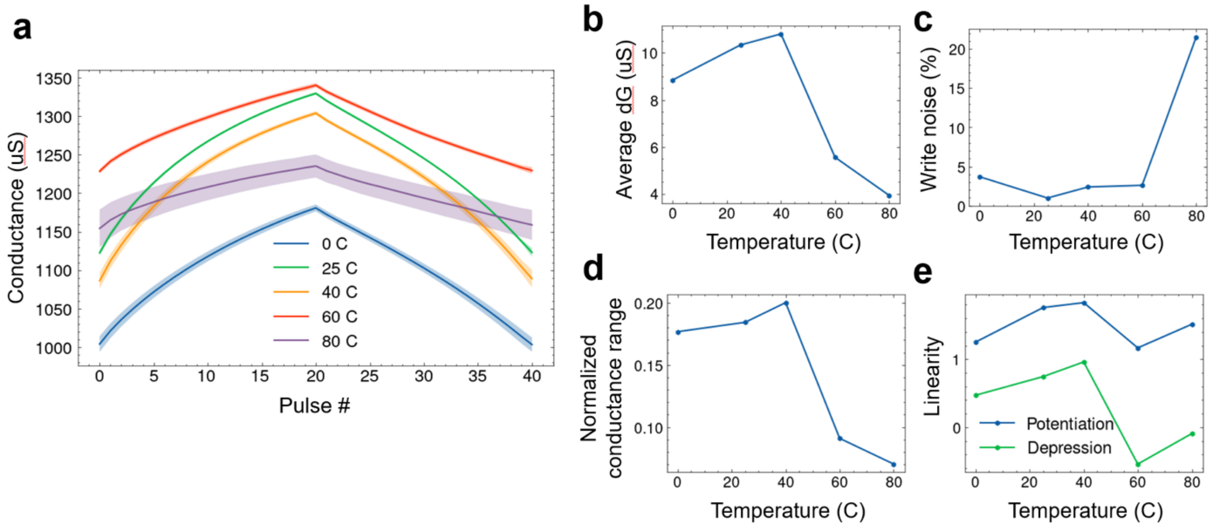

Supplementary Figure 13. a, mBLAST performance at various temperatures for **b**, average change in conductance, **c**, write noise, **d**, normalized conductance range, and **e**, linearity. There is a clear change in performance between 40°C and 60°C in all metrics evaluated.

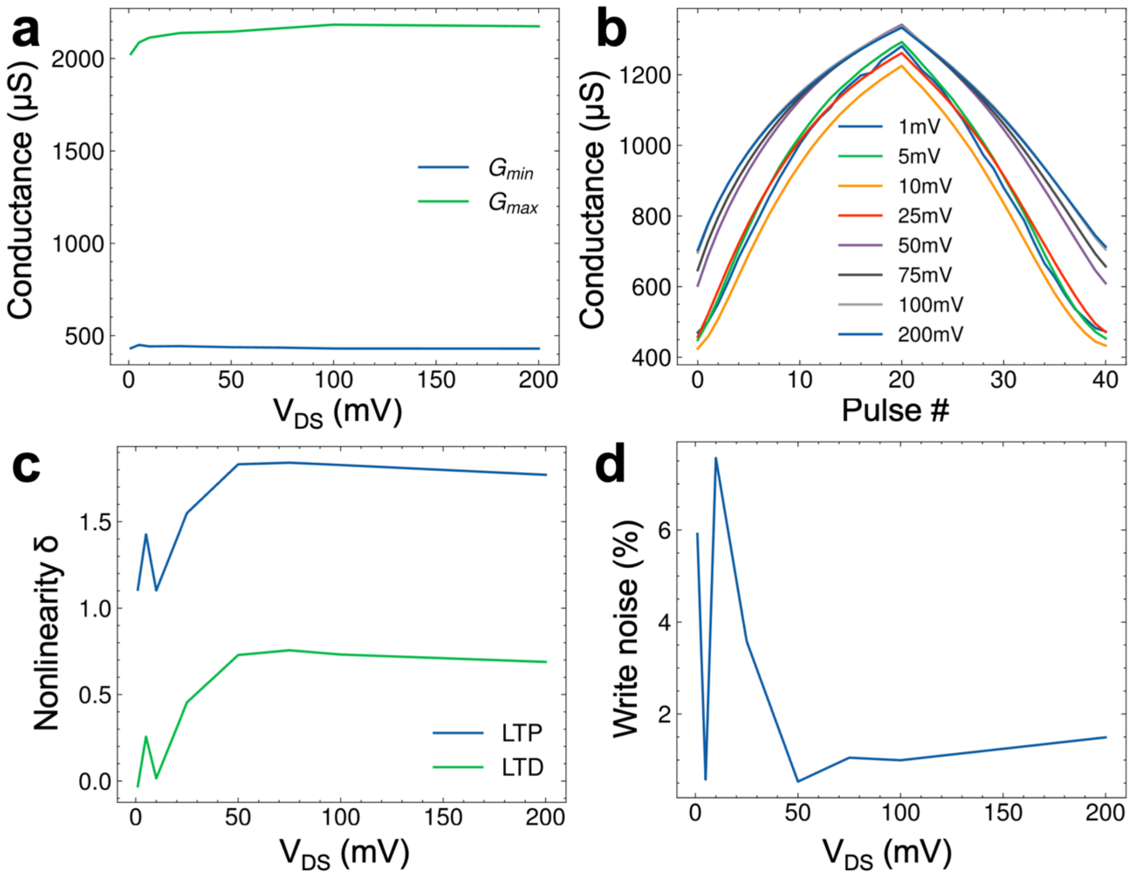

Supplementary Figure 14. mBLAST performance at various read voltage V_{DS} for **a**, conductance, **b**, pulse sweeps, **c**, nonlinearity, and **d**, write noise. There is some variability in performance for all presented metrics at voltages below 50 mV which stabilizes above 50 mV. This is most likely due to the read noise at low voltage.

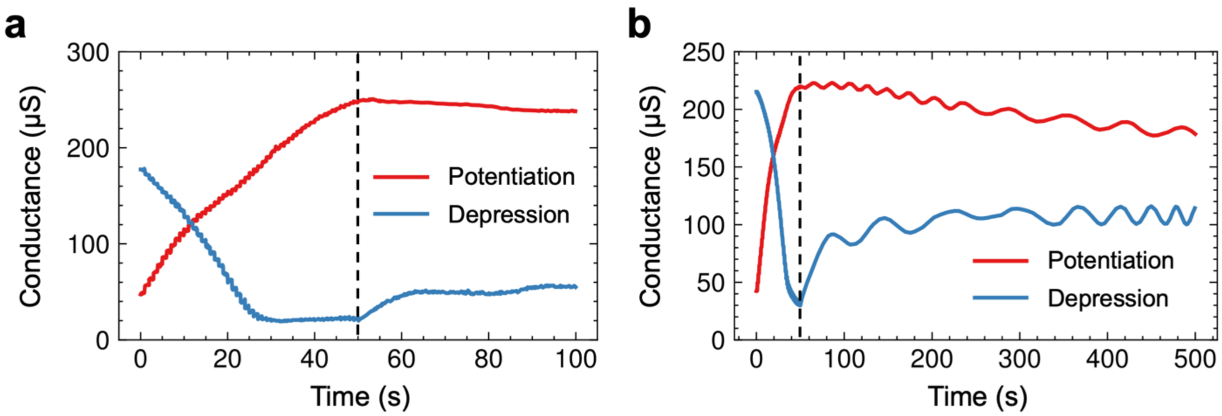

Supplementary Figure 15. Retention tests of two μBLAST devices in **a** and **b**, respectively, both with channel dimensions of 40 μm by 10 μm. The device in **a** is sampled for 50 seconds after a series of 50 current pulses are delivered for 50 seconds while the device in **b** is sampled for 450 seconds. Regime to the left of the dotted black line indicates where pulses are applied.

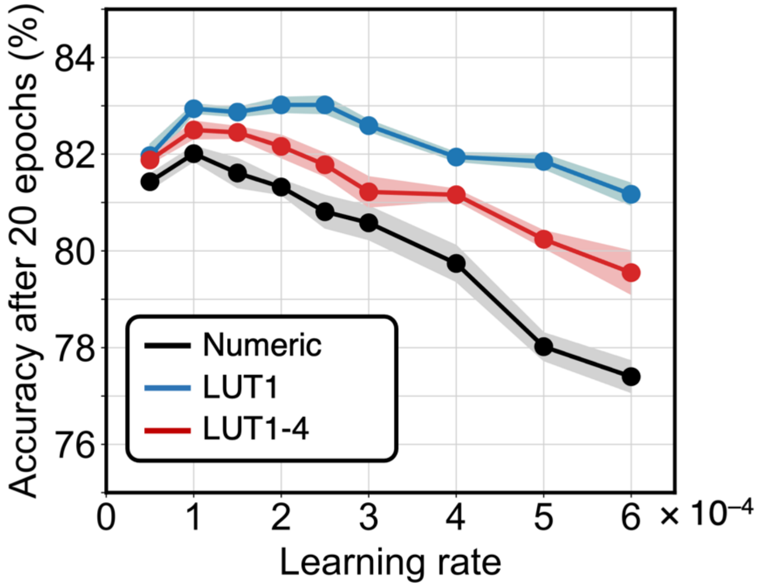

Supplementary Figure 16. Learning rate sweep confirming that the training benefit for μBLAST synapses is not limited to specific learning rates.

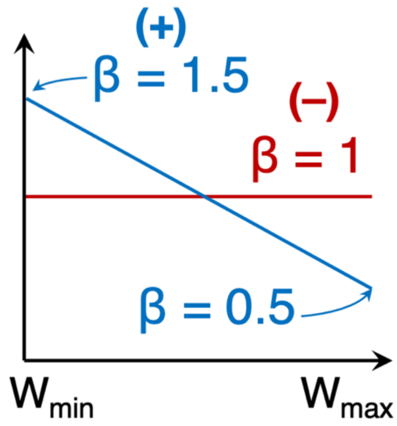

Supplementary Figure 17. Graphical representation of the modified update scheme for the numerical metaplastic weights. The computed optimizer update is multiplied by β, emulating the synaptic characteristics of μBLASTs. β for the positive (negative) update is shown in blue (red).

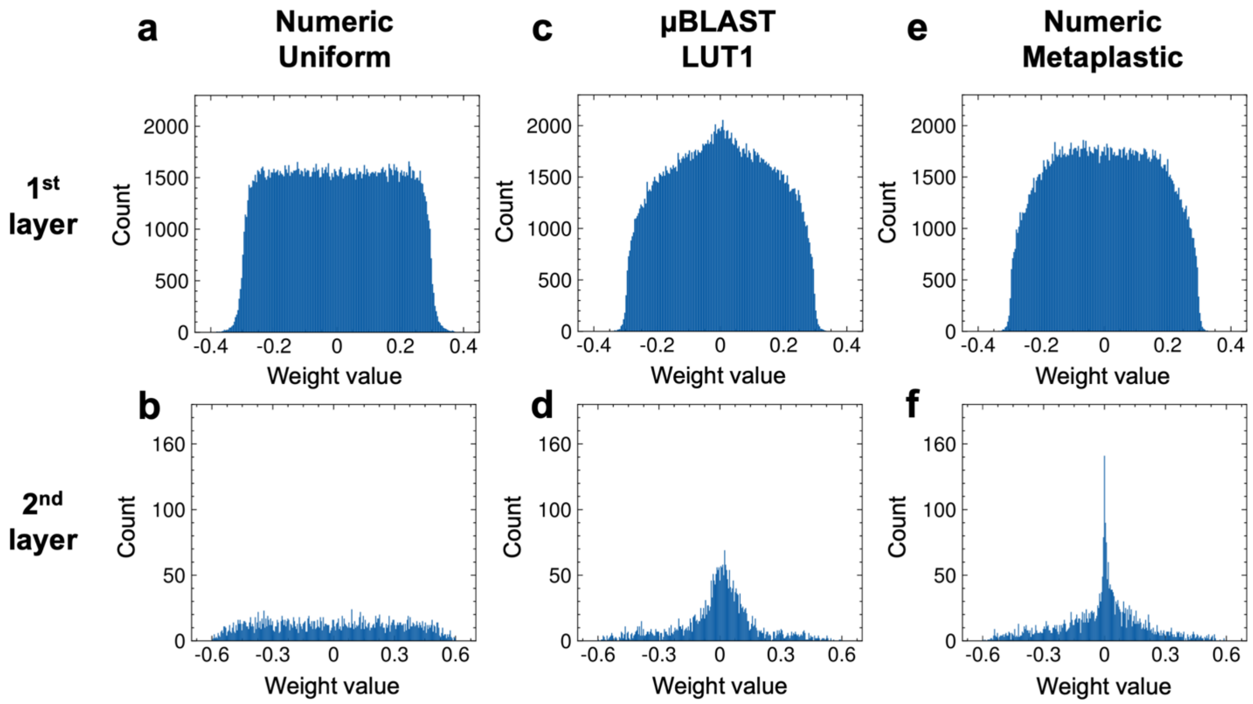

Supplementary Figure 18. Histogram representation of weight distributions for **a, b,** numeric uniform, **c, d,** μBLASTs, and **e, f,** numeric metaplastic, for the 1st and 2nd layer, respectively. The shape of the weight distribution for μBLASTs qualitatively matches that of the numeric metaplastic weights, confirming a weight normalization effect.

Supplementary References

1. Y. van de Burgt, E. Lubberman, E. J. Fuller, S. T. Keene, G. C. Faria, S. Agarwal, M. J. Marinella, A. Alec Talin & A. Salleo. A non-volatile organic electrochemical device as a low-voltage artificial synapse for neuromorphic computing. *Nature Materials* **16,** 414–418 (2017).
2. Y. Li, T. P. Xiao, C. H. Bennett, E. Isele, A. Melianas, H. Tao, M. J. Marinella, A. Salleo, E. J. Fuller & A. A. Talin. In situ Parallel Training of Analog Neural Network Using Electrochemical Random-Access Memory. *Frontiers in Neuroscience* **15,** (2021).
3. Q. Lai, L. Zhang, Z. Li, W. F. Stickle, R. S. Williams & Y. Chen. Ionic/Electronic Hybrid Materials Integrated in a Synaptic Transistor with Signal Processing and Learning Functions. *Advanced Materials* **22,** 2448–2453 (2010).
4. C. He, J. Tang, D.-S. Shang, J. Tang, Y. Xi, S. Wang, N. Li, Q. Zhang, J.-K. Lu, Z. Wei, Q. Wang, C. Shen, J. Li, S. Shen, J. Shen, R. Yang, D. Shi, H. Wu, S. Wang, *et al.* Artificial Synapse Based on van der Waals Heterostructures with Tunable Synaptic Functions for Neuromorphic Computing. *ACS Applied Materials & Interfaces* **12,** 11945–11954 (2020).
5. H. Tian, Q. Guo, Y. Xie, H. Zhao, C. Li, J. J. Cha, F. Xia & H. Wang. Anisotropic Black Phosphorus Synaptic Device for Neuromorphic Applications. *Advanced Materials* **28,** 4991–4997 (2016).
6. J. Yu, X. Yang, G. Gao, Y. Xiong, Y. Wang, J. Han, Y. Chen, H. Zhang, Q. Sun & Z. L. Wang. Bioinspired mechano-photonic artificial synapse based on graphene/MoS_2 heterostructure. *Science Advances* **7,** (2021).
7. S. Wang, C. Chen, Z. Yu, Y. He, X. Chen, Q. Wan, Y. Shi, D. W. Zhang, H. Zhou, X. Wang & P. Zhou. A MoS_2/PTCDA Hybrid Heterojunction Synapse with Efficient Photoelectric Dual Modulation and Versatility. *Advanced Materials* **31,** 1806227 (2019).
8. T. Paul, T. Ahmed, K. Kanhaiya Tiwari, C. Singh Thakur & A. Ghosh. A high-performance MoS_2 synaptic device with floating gate engineering for neuromorphic computing. *2D Materials* **6,** 045008 (2019).
9. M. T. Sharbati, Y. Du, J. Torres, N. D. Ardolino, M. Yun & F. Xiong. Low-Power, Electrochemically Tunable Graphene Synapses for Neuromorphic Computing. *Advanced Materials* **30,** (2018).
10. J. Zhu, Y. Yang, R. Jia, Z. Liang, W. Zhu, Z. U. Rehman, L. Bao, X. Zhang, Y. Cai, L. Song & R. Huang. Ion Gated Synaptic Transistors Based on 2D van der Waals Crystals with Tunable Diffusive Dynamics. *Advanced Materials* **30,** 1800195 (2018).
11. C. Yang, D. Shang, N. Liu, E. J. Fuller, S. Agrawal, A. A. Talin, Y. Li, B. Shen & Y. Sun. All-Solid-State Synaptic Transistor with Ultralow Conductance for Neuromorphic Computing. *Advanced Functional Materials* **28,** 1804170 (2018).
12. X. Zhu, D. Li, X. Liang & W. D. Lu. Ionic modulation and ionic coupling effects in MoS2 devices for neuromorphic computing. *Nature Materials* **18,** 141–148 (2019).
13. J.-T. Yang, C. Ge, J.-Y. Du, H.-Y. Huang, M. He, C. Wang, H.-B. Lu, G.-Z. Yang & K.-J. Jin. Artificial Synapses Emulated by an Electrolyte-Gated Tungsten-Oxide Transistor. *Advanced Materials* **30,** 1801548 (2018).
14. W. Hu, J. Jiang, D. Xie, B. Liu, J. Yang & J. He. Proton–electron-coupled MoS_2 synaptic transistors with a natural renewable biopolymer neurotransmitter for brain-inspired neuromorphic learning. *Journal of Materials Chemistry C* **7,** 682–691 (2019).

15. C. Y. Han, Y. Li, Z. X. Zhang, W. H. Liu, X. Li, X. Guo, J. Wan, G. H. Zhang, X. L. Wang & Q. Lin. An artifical synapse based on graphene field-effect transistor with silver gel/polarized-aptamer gate. *Organic Electronics* **92,** 106118 (2021).
16. W. Qin, B. H. Kang & H. J. Kim. Flexible Artificial Synapses with a Biocompatible Maltose–Ascorbic Acid Electrolyte Gate for Neuromorphic Computing. *ACS Applied Materials & Interfaces* **13,** 34597–34604 (2021).
17. L. Hu, L. Li, K. Chang, X. Lin, P. Huang & S. Zhang. Ultrasensitive Freestanding and Mechanically Durable Artificial Synapse with Attojoule Power Based on Na-Salt Doped Polymer for Biocompatible Neuromorphic Interface. *Advanced Functional Materials* **31,** 2106015 (2021).
18. H. Wei, R. Shi, L. Sun, H. Yu, J. Gong, C. Liu, Z. Xu, Y. Ni, J. Xu & W. Xu. Mimicking efferent nerves using a graphdiyne-based artificial synapse with multiple ion diffusion dynamics. *Nature Communications* **12,** 1068 (2021).
19. T. Hussain, H. Abbas, C. Youn, H. Lee, T. Boynazarov, B. Ku, Y. Jeon, H. Han, J. H. Lee, C. Choi & T. Choi. Cellulose Nanocrystal Based Bio-Memristor as a Green Artificial Synaptic Device for Neuromorphic Computing Applications. *Advanced Materials Technologies* **7,** 2100744 (2022).
20. H. Wang, W. Lu, S. Hou, B. Yu, Z. Zhou, Y. Xue, R. Guo, S. Wang, K. Zeng & X. Yan. A 2D-SnSe film with ferroelectricity and its bio-realistic synapse application. *Nanoscale* **12,** 21913–21922 (2020).
21. M. Wang, S. Cai, C. Pan, C. Wang, X. Lian, Y. Zhuo, K. Xu, T. Cao, X. Pan, B. Wang, S.-J. Liang, J. J. Yang, P. Wang & F. Miao. Robust memristors based on layered two-dimensional materials. *Nature Electronics* **1,** 130–136 (2018).
22. X. Yan, C. Qin, C. Lu, J. Zhao, R. Zhao, D. Ren, Z. Zhou, H. Wang, J. Wang, L. Zhang, X. Li, Y. Pei, G. Wang, Q. Zhao, K. Wang, Z. Xiao & H. Li. Robust Ag/ZrO_2/WS_2/Pt Memristor for Neuromorphic Computing. *ACS Applied Materials & Interfaces* **11,** 48029–48038 (2019).
23. R. Xu, H. Jang, M.-H. Lee, D. Amanov, Y. Cho, H. Kim, S. Park, H. Shin & D. Ham. Vertical MoS_2 Double-Layer Memristor with Electrochemical Metallization as an Atomic-Scale Synapse with Switching Thresholds Approaching 100 mV. *Nano Letters* **19,** 2411–2417 (2019).
24. Y. Kim, C. H. Park, J. S. An, S.-H. Choi & T. W. Kim. Biocompatible artificial synapses based on a zein active layer obtained from maize for neuromorphic computing. *Scientific Reports* **11,** 20633 (2021).
25. B. Tian, L. Liu, M. Yan, J. Wang, Q. Zhao, N. Zhong, P. Xiang, L. Sun, H. Peng, H. Shen, T. Lin, B. Dkhil, X. Meng, J. Chu, X. Tang & C. Duan. A Robust Artificial Synapse Based on Organic Ferroelectric Polymer. *Advanced Electronic Materials* **5,** 1800600 (2019).
26. Y. Chen, Y. Zhou, F. Zhuge, B. Tian, M. Yan, Y. Li, Y. He & X. S. Miao. Graphene–ferroelectric transistors as complementary synapses for supervised learning in spiking neural network. *npj 2D Materials and Applications* **3,** 31 (2019).
27. J. Gao, Y. Zheng, W. Yu, Y. Wang, T. Jin, X. Pan, K. P. Loh & W. Chen. Intrinsic polarization coupling in 2D α-In_2Se_3 toward artificial synapse with multimode operations. *SmartMat* **2,** 88–98 (2021).
28. T. F. Schranghamer, A. Oberoi & S. Das. Graphene memristive synapses for high precision neuromorphic computing. *Nature Communications* **11,** (2020).

29. X. Sun & S. Yu. Impact of Non-Ideal Characteristics of Resistive Synaptic Devices on Implementing Convolutional Neural Networks. *IEEE Journal on Emerging and Selected Topics in Circuits and Systems* **9,** 570–579 (2019).
