# Supplementary figures and images for: Metaplastic and energy-efficient biocompatible graphene artificial synaptic transistors for enhanced accuracy neuromorphic computing
Source: Nat Commun. 2022 Jul 28;13:4386. doi: 10.1038/s41467-022-32078-6 (PMC9334620; doi:10.1038/s41467-022-32078-6)

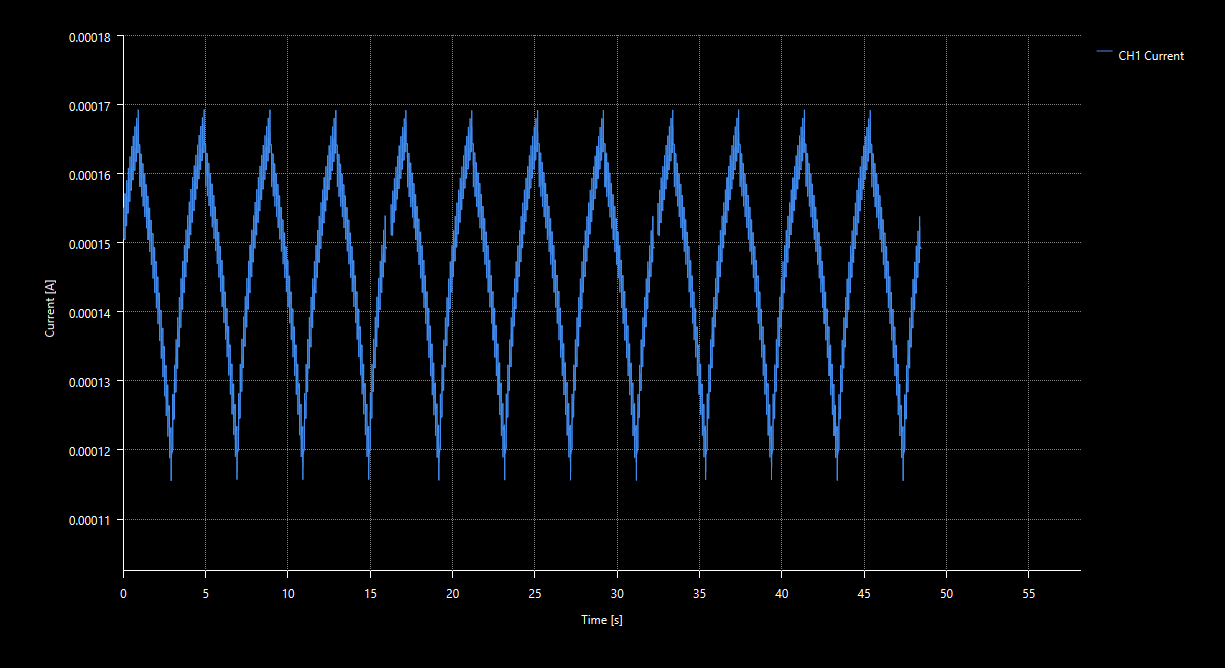

Supplement: Supplementary file 2 — Source Data [file 41467_2022_32078_MOESM2_ESM.zip › NatComm_figdata/Fig1f/210914_2Lhole3_10uA_multiramp.png]

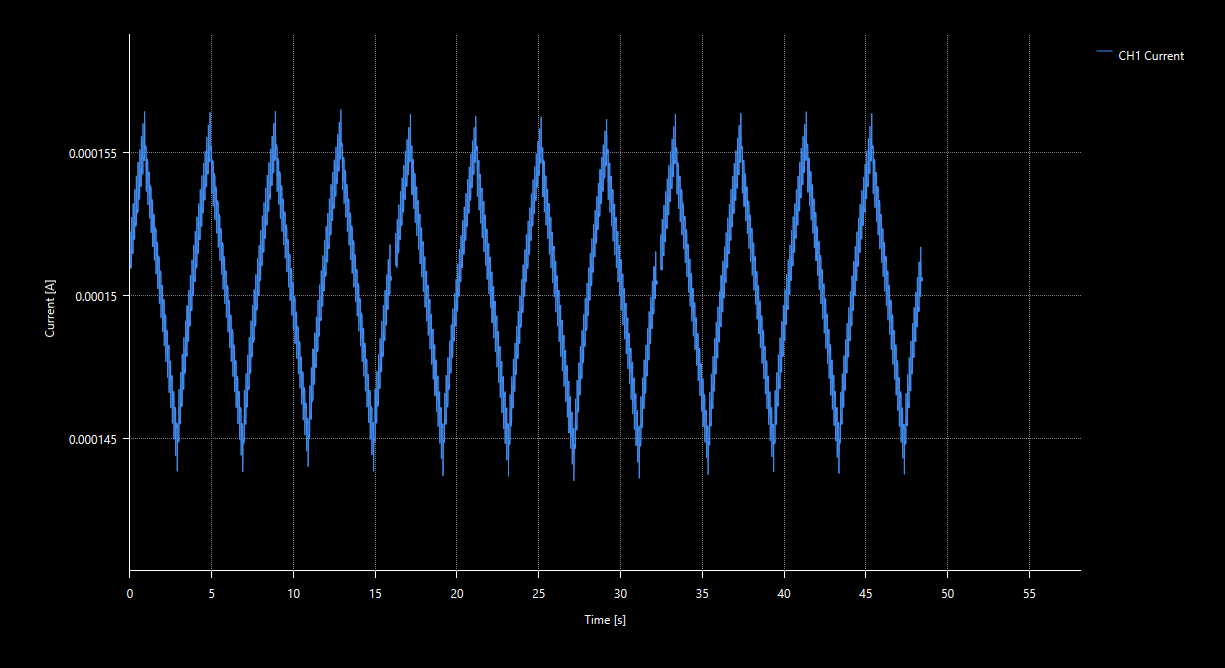

Supplement: Supplementary file 2 — Source Data [file 41467_2022_32078_MOESM2_ESM.zip › NatComm_figdata/Fig1f/210914_2Lhole3_2.5uA_multiramp.png]

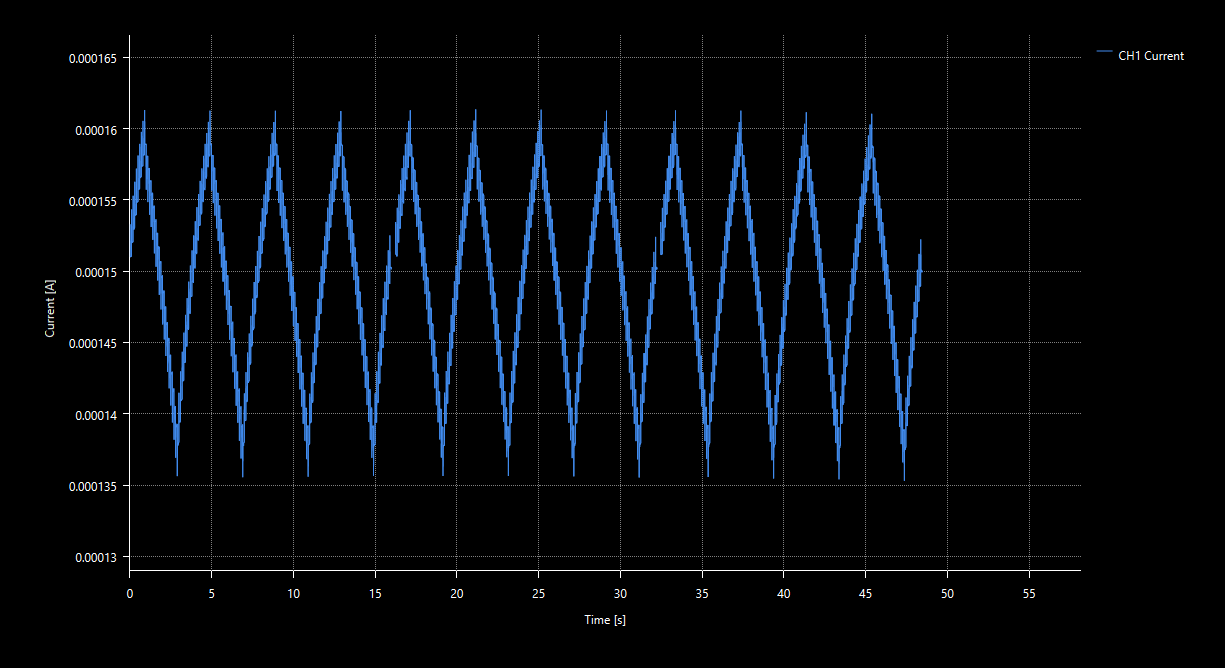

Supplement: Supplementary file 2 — Source Data [file 41467_2022_32078_MOESM2_ESM.zip › NatComm_figdata/Fig1f/210914_2Lhole3_5uA_multiramp.png]

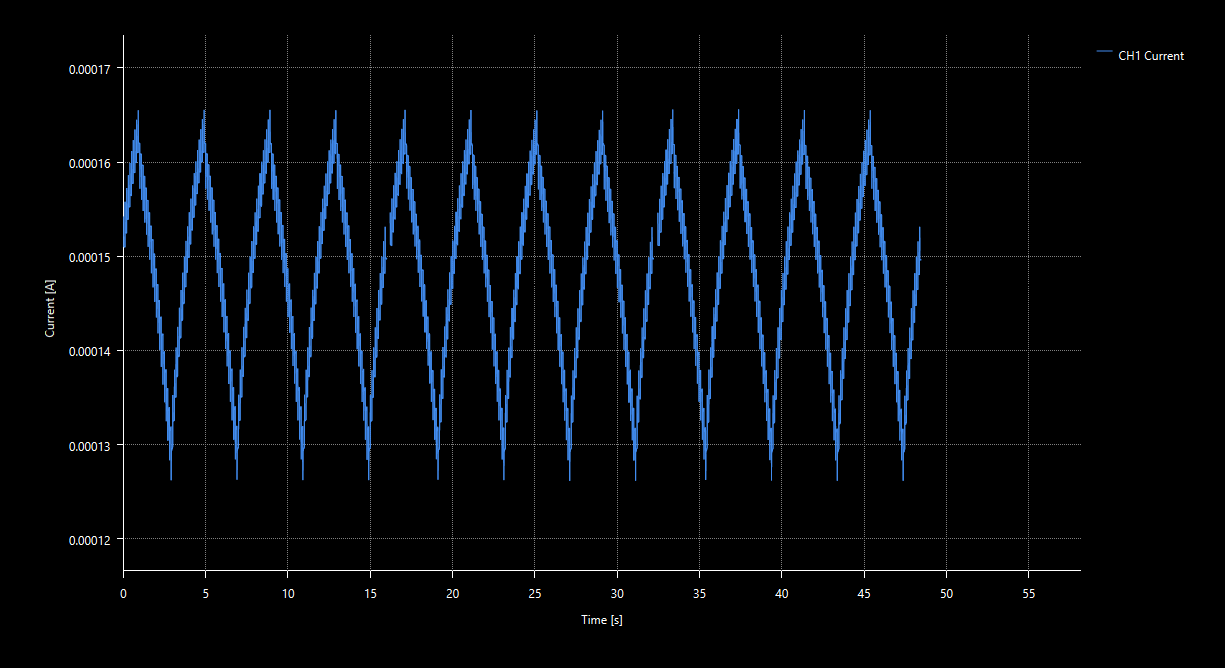

Supplement: Supplementary file 2 — Source Data [file 41467_2022_32078_MOESM2_ESM.zip › NatComm_figdata/Fig1f/210914_2Lhole3_7.5uA_multiramp.png]

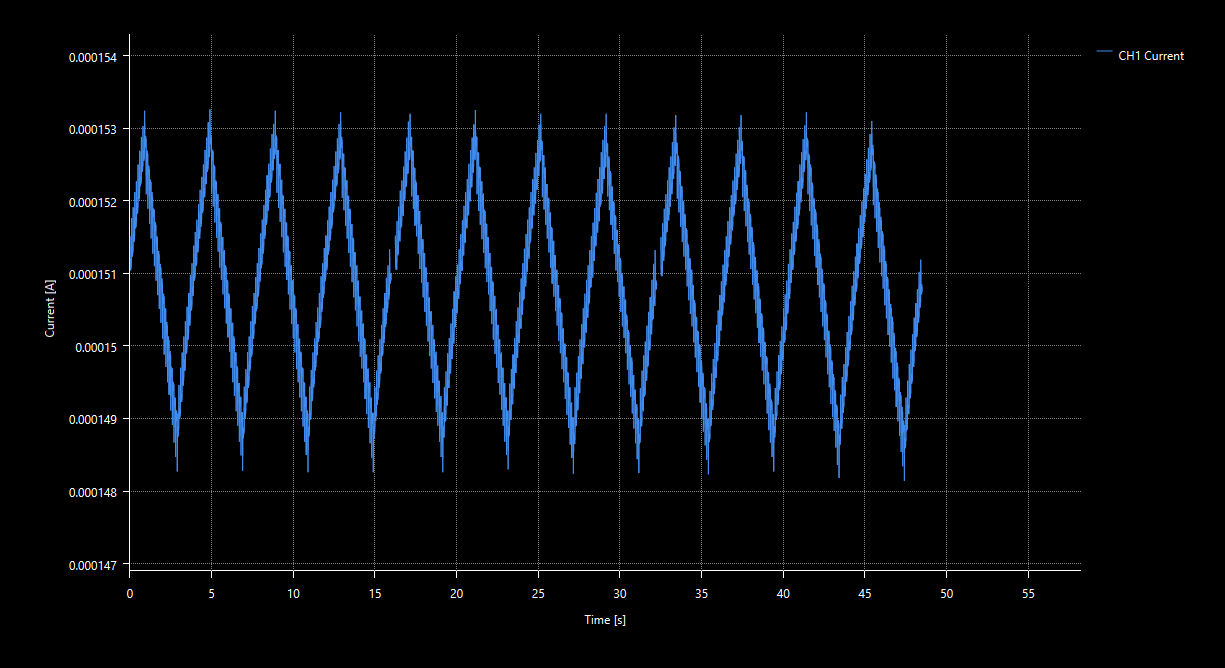

Supplement: Supplementary file 2 — Source Data [file 41467_2022_32078_MOESM2_ESM.zip › NatComm_figdata/Fig1f/210914_2Lhole3_1uA_multiramp.png]
